# An Expert Perspective on a New Recombinant Pertussis-Only Vaccine and Its Potential Clinical and Programmatic Advantages in Australia, New Zealand, and Beyond

**DOI:** 10.3390/vaccines14090736

**Published:** 2026-08-26

**Authors:** Peter McIntyre, Anita H. J. van den Biggelaar, Frank Beard, Mary Nowlan, Annette K. Regan, Rodney Pearce, Terry Nolan, Catherine Hughes, Peter Richmond

**Affiliations:** 1 Department of Women’s and Children’s Health, School of Medicine, University of Otago, 362 Leith Street, North Dunedin, Dunedin 9016, New Zealand; peter.mcintyre@otago.ac.nz; 2 Wesfarmers Centre of Vaccines and Infectious Diseases, The Kids Research Institute Australia, 15 Hospital Avenue, Nedlands, WA 6009, Australia; peter.richmond@uwa.edu.au; 3 BioNet, 19 Soi Udomsuk 37, Sukhumvit 103 Road, Bangjak, Prakanong, Bangkok 10260, Thailand; 4 School of Public Health, Faculty of Medicine and Health, The University of Sydney, 1 Western Avenue, Camperdown, NSW 2050, Australia; frank.beard@sydney.edu.au; 5 Immunisation Advisory Centre, Department of General Practice and Health Care, The University of Auckland, Private Bag 92019, Victoria St West, Auckland 1142, New Zealand; m.nowlan@auckland.ac.nz; 6 Department of Epidemiology, Fielding School of Public Health, University of California Los Angeles, 650 Charles E Young Dr S, Los Angeles, CA 90095, USA; annette.k.regan@kp.org; 7 Medical HQ Glynde, 127 Glynburn Road, Adelaide, SA 5070, Australia; drpearce@medicalhq.com.au; 8 Peter Doherty Institute for Infection and Immunity, The University of Melbourne, Parkville, VIC 3010, Australia; t.nolan@unimelb.edu.au; 9 Immunisation Foundation of Australia, P.O. Box 186, Bassendean, WA 6935, Australia; catherine@ifa.org.au; 10 School of Medicine, The University of Western Australia, 35 Stirling Highway, Crawley, WA 6009, Australia

**Keywords:** pertussis, booster, vaccination, pertussis-only, recombinant

## Abstract

Despite high childhood coverage and booster doses of pertussis vaccination across the life course, pertussis (whooping cough) outbreaks continue to occur, including in Australia and New Zealand. In January 2026, the European Medicines Agency (EMA) granted marketing authorisation to a new pertussis-only booster vaccine (VacPertagen^TM^): the first time in two decades that a new acellular pertussis vaccine has been approved in the European Union. Anticipating future licensure of the vaccine in Australia and New Zealand, an expert panel was convened to discuss the clinical and programmatic advantages that this new vaccine may offer in the context of current pertussis epidemiology and vaccination strategies inSuch advantages may also be applicable to other high-income countries experiencing ongoing pertussis outbreaks.

## 1. Pertussis in Australia and New Zealand

Many countries have reported pertussis outbreaks post-COVID-19, following low case numbers during the pandemic when public health and social measures decreased respiratory transmission in the community [1]. In Australia, a record breaking > 57,000 pertussis notifications were reported in 2024 [1,2]. Even before the pandemic, 5–14-year-olds represented a large proportion of pertussis notifications, but this increased markedly from 41.4% of notifications in 2019 to 57.5% in 2024. No less than 1% of Australian children in this age group had notified pertussis in 2024 (notification rate 1003 per 100,000) [1,2]. The next highest notification rate was in 1–4-year-old children, at 409 reported cases per 100,000. Notably, notifications remained low in infants < 12 months of age, which is consistent with ongoing effectiveness of maternal pertussis immunisation programs (offered to women each pregnancy free of charge) and the primary infant immunisation series [3]. In Australia, the pertussis immunisation schedule has a total of six childhood doses, including booster doses at 18 months, 4 years and 12–13 years of age after a primary vaccination series at 2, 4, and 6 months of age. Despite trends of decreasing coverage for both maternal and childhood pertussis vaccines since 2020, vaccination rates remain relatively high in Australia (>90% coverage for dose 4 or 5; >70% coverage for adolescent dose; and >70% for pregnancy vaccination) [4,5,6]. Lack of vaccination uptake therefore does not explain the ongoing pertussis outbreaks.

In New Zealand the situation is different. Here, pertussis cases started to increase in October 2024, but differently from Australia notifications rates are the highest in infants < 12 months of age [7,8]. The New Zealand pertussis immunisation schedule includes five childhood doses (a toddler booster dose is not included), a dose for pregnant individuals each pregnancy, and one dose to everyone from the age of 65; however, vaccination rates are suboptimal, especially among Indigenous Māori and Pacific people and those living in areas of highest deprivation [9]. A continued low uptake of maternal pertussis immunisation and delayed childhood vaccinations have left infants at high risk of severe disease now that pertussis circulation has risen. Most infants < 12 months old who are hospitalised for pertussis are Māori, and in almost all cases the infants’ mothers have not received pertussis vaccination during pregnancy [9]. Of note, the lower rate of pertussis notifications in individuals older than 12 months is likely an underestimation of the true burden: although pertussis is a notifiable disease in New Zealand, non-hospitalised cases do no often get tested and reported [10].

The interpretation of epidemiologic trends across countries is complex, but data from Australia and comparable countries suggest that current acellular pertussis vaccines—despite multiple booster doses—do not offer full or long-lasting protection. Several factors may contribute to this, including waning vaccine immunity, lesser vaccine effectiveness against mild disease and transmission than against severe illness [11,12]. It is clear that new vaccines offering higher and more sustained protection would be beneficial and preferential over increasing the number or frequency of booster doses.

## 2. A New Pertussis Vaccine—What Is Different?

A new vaccine containing recombinant pertussis toxin (PT, 5 µg) and filamentous haemagglutinin (FHA, 5 µg) was licensed in January 2026 in the European Union (EU) as VacPertagen^TM^ (BioNet Europe, Lyon, France) for individuals aged 12 years and older, including pregnant women [13]. The vaccine is also marketed as Pertagen^®^ (BioNet Asia, Bangkok, Thailand) in Thailand and Singapore, where it is approved for booster vaccination of individuals aged 3 years and older, including pregnant women. VacPertagen differs from tetanus-diphtheria-acellular pertussis booster vaccines (Tdap) used for over two decades in New Zealand, Australia and many other high-income countries in two important ways: (1) it contains PT that has been detoxified using recombinant technologies rather than chemical processes and (2) it is not combined with tetanus or diphtheria toxoid vaccines (it is pertussis-only).

### 2.1. Pertussis Toxin-Neutralising Antibodies Are the Key Mechanism of Protection Conferred by Acellular Pertussis Vaccination

Pertussis toxin (PT) is the most important component of acellular pertussis vaccines [14]. While the number of *Bordetella pertussis* antigens contained in acellular pertussis vaccines may vary, all vaccine formulations contain PT with the aim to induce high titers of neutralising antibodies to mitigate disease symptoms. Vaccination with mono-component acellular pertussis vaccine (containing only PT and no other pertussis antigens) is sufficient to confer protection at levels similar to multicomponent vaccines, as is evident in Denmark where a PT-only pertussis vaccine was effectively used for many years to vaccinate children and adults, and more recently also pregnant women [15,16].

PT plays a central role in disease severity by actions including impairing immune function and inducing many of the systemic manifestations of infection [17,18]. Suppression of the early immune response allows *B. pertussis* bacteria to colonise the respiratory tract and multiply. PT is the dominant driver of inflammation that can lead to severe, uncontrollable (paroxysmal) coughing fits and in the most severe cases can cause broken ribs, pneumonia, and brain damage from lack of oxygen. In infants unprotected by maternal or post-natal immunisation, very high (pseudo-leukemic) white cell counts can lead to intravascular sludging, pulmonary hypertension, respiratory failure and death. The amount of secreted PT correlates with increased clinical severity, increased rates of hospitalisation, and death [18,19,20,21]. Inherently, the ability to neutralise PT is a critical component of protective immunity that depends on both the quantity and quality of functional anti-PT antibodies [22]. PT-neutralising antibodies are necessary and sufficient to block disease symptoms, as demonstrated in experimental baboon infection models [23,24],. While no correlate of protection has been established, higher anti-PT antibody titers are correlated with less severe disease in humans [25,26], and the effectiveness of pertussis booster vaccination declines in parallel with the waning of anti-PT antibodies [27,28], which further support the central role of anti-PT antibodies mediating protection. Since all antibodies inevitably wane but remain elevated at critical levels for a longer period when initial titers are higher, the aim of acellular pertussis vaccination should be to induce high titers of anti-PT antibodies that have a high capacity of binding and neutralising PT and that persist at sufficiently high levels for a prolonged period. As we discuss in the next section, the method that is used to inactivate PT in acellular pertussis vaccines so that it can be safely administered to humans impacts the anti-PT antibody response and capacity to neutralise PT. With respect to other pertussis antigens that may be variably included in acellular pertussis vaccines, their contribution in conferring protection is still under discussion and needs further study [14]. Co-administration of filamentous hemagglutinin (FHA) with PT was found to have an enhancing protective effect in mouse challenge models [29,30]. Since the effectiveness of acellular pertussis vaccines is similar against pertactin (PRN)-positive and PRN-negative strains, it is debatable if the antibody response to PRN necessarily contributes to protection against pertussis [31]. Serum levels of anti-fimbriae (FIM) antibodies have been correlated with protection against household exposure to *B. pertussis*, but only some of the currently used acellular pertussis vaccines contain FIM (which is a mixture of both Fim 2 and Fim3) [25].

### 2.2. Chemical Versus Genetic Detoxification of PT: How Does It Differ?

Traditionally, detoxification of PT has been achieved using chemical treatment (commonly formaldehyde or glutaraldehyde). While this effectively inactivates toxic properties, it causes varying degrees of denaturation with changes in conformational epitopes, leading to loss of protection [32]. Genetic detoxification uses recombinant technologies to introduce specific point mutations that knock out PT’s enzymatic activity while preserving protein configuration and conformational epitopes associated with highly PT neutralising immune responses. In VacPertagen, PT is genetically detoxified by substitution of two amino acids in its S1 subunit that knocks-out the ADP-ribosyl transferase activity of the toxin [33]. Data obtained by crystallography show that the PT antigen included in VacPertagen has an identical structure to wild-type PT [34]; this includes the preservation of conformational epitopes recognised by two potent neutralising anti-PT monoclonal antibodies, hu11E6 and hu1B7, that were shown to passively protect baboons against a lethal challenge with *B. pertussis* [23,34]. As summarised in the next section, due to containing genetically detoxified PT, VacPertagen confers stronger and longer lasting functional anti-PT antibody responses than the currently used chemically detoxified Tdap vaccines.

### 2.3. Comparative Immunogenicity of Vaccines Containing Genetically Versus Chemically Detoxified Pertussis Toxin

Data from randomised controlled clinical trials in adolescents, adults and pregnant women consistently show that vaccination with genetically detoxified PT improves anti-PT antibody responses, including higher titres, higher antibody avidity, and higher functional neutralisation. Whereas pertussis vaccination studies generally have only reported on levels of anti-PT binding antibodies, reports from clinical trials involving VacPertagen all include results on anti-PT neutralising antibody titers in addition to antibody binding titers. Table 1 summarises clinical trial results for PT neutralising antibody titres 1 month post-vaccination: across different populations, PT-neutralising antibody titres were consistently 6.5-to-8.5-fold or 2-to-2.5-fold higher after vaccination with VacPertagen compared with licensed 3-component or 5-component acellular pertussis vaccine, respectively [13,35,36,37,38]. In studies of maternal immunisation in the second or third trimester, titres of maternally derived PT-neutralising antibodies were found to be 2-to-10-fold higher in infants at birth and remained significantly higher at 2 months [13,39,40]. Further qualitative analysis has also demonstrated that antibodies induced by maternal vaccination with genetically inactivated PT and transferred to infants are of higher avidity [40].

The induction of higher anti-PT antibody titres subsequently also leads to longer persistence of elevated antibody titres. Follow-up studies in adults 3 years post-booster and in adolescents 5 years post-booster have found that levels of PT neutralising antibodies remained 8-fold and 2.5-fold higher compared to baseline levels, respectively, whereas following booster vaccination with a licensed Tdap vaccine, neutralising antibodies had returned to pre-vaccination levels after 2–3 years (see Table 1) [13,41,42]. Notably, the more rapid waning of anti-PT antibodies observed with chemically inactivated acellular pertussis booster vaccines correlates with the short-lived effectiveness of these vaccines observed in post-licensure effectiveness studies [11,43]. While a correlate of protection has not been established, one may assume that the persistence of PT-neutralising antibody titres at elevated levels is ultimately associated with persistent protection. Future post-implementation studies will show if the longer-lasting pertussis-neutralising antibody responses induced by recombinant acellular pertussis vaccine translate into a longer duration of protection.

**Table 1:** Immunogenicity assessed as PT-neutralising antibody titres 1 month post-vaccination and persistence at 1, 3 and 5 years post-vaccination with recombinant acellular pertussis vaccine or traditional Tdap vaccine in different study populations.

	PT-Neutralising Antibody Titres Geometric Mean Titre (95% Confidence Interval)	
1 Month Post-Vaccination	Recombinant Acellular Pertussis Vaccine	Traditional Tdap Vaccine
Adolescents (12–17 yrs old) [13,35]	276 (182–419) IU/mL d	36 (26–51) IU/mL a
Adolescents (11–16 yrs old) [36]	128 (97–169) IU/mL c,d	74 (50–110) IU/mL b
Adults (18–75 yrs old) [13]	253 (181–354) IU/mL d	30 (21–41) IU/mL a
Adults (18–30 yrs old) [38]	147 (108–200) IU/mL d	69 (50–96) IU/mL b
Pregnant women (18–40 yrs old) [13]	207 (91–469) IU/mL d	32 (20–53) IU/mL a
1 year post-vaccination		
Adolescents (12–17 yrs old) [13,35]	77 (53–112) d	12 (9–17) a
Adolescents (11–16 yrs old) [36]	37 (27–50) c	25 (18–36) b
Adults (18–30 yrs old) [38]	31 (21–46) d	16 (11–22) b
3 years post-vaccination		
Adolescents (12–17 yrs old) [42]	48 (24–92) d	15 (9–24) a
Adults (18–75 yrs old) [13]	46 (33–64) d	9 (7–12) a
5 years post-vaccination		
Adolescents (12–17 yrs old) [13,41]	26 (13–52) d	12 (7–19) a

Age of the study population is age at the time of vaccination (not at follow-up). Comparator Tdap vaccine containing (a) 2.5 µg or (b) 8 µg chemically detoxified PT. (c) Participants were co-administered tetanus and diphtheria vaccines. (d) Antibody levels were significantly higher in the recombinant acellular pertussis vaccine group.

### 2.4. The Role of Cellular Immunity

Protection by acellular pertussis vaccines is primarily mediated by antibodies against PT. This is particularly true for passive immune protection in newborns where cellular immunity plays no role. In the case of active immunisation, the induction of memory B-cell responses may contribute to longer-lived immune protection.

Soluble antigens as contained in all acellular pertussis vaccines are exquisite T-cell dependent antigens that are able to induce antibodies only thanks to the help provided by CD4^+^ T cells. Whilst no data are available for VacPertagen, studies with formerly available paediatric acellular pertussis vaccine containing genetically inactivated PT demonstrated the activation of CD4^+^ T-helper cell immunity of a mixed Th1-Th2-type phenotype [44], which is characteristic of vaccination with soluble vaccine antigens formulated with aluminum salts. Soluble antigens plus alum are unable to induce CD8^+^ cytotoxic cells.

A clinical trial involving adolescents in Switzerland has reported that booster vaccination with VacPertagen induced PT-specific memory B cells that persisted 1 year later, while in contrast no significant induction of memory B-cells was observed after booster vaccination with the Tdap comparator [36]. Similar results were reported for a Phase 1 trial with another candidate recombinant acellular pertussis vaccine containing genetically detoxified PT [45].

## 3. Potential Use Cases for a Recombinant Pertussis-Only Vaccine

### 3.1. Maternal Immunisation

If maternal immunisation with recombinant pertussis vaccine delivers more durable protection in infants than current Tdap booster vaccines, this could be particularly beneficial if receipt of infant pertussis-containing vaccines is delayed—as seen in Māori and Pacific people in New Zealand—even at suboptimal levels of maternal vaccination coverage. Longer persistence of protective anti-PT neutralising antibodies in vaccinated women could also provide greater protection for infants born to multiparous pregnant women who do not receive repeat boosters for subsequent pregnancies. It is well documented that maternal vaccination uptake decreases with subsequent pregnancies, especially if closely spaced [46,47,48], with documented instances of death from pertussis in newborns whose mothers received Tdap vaccine in a previous but not the current pregnancy. Confirmatory data from post-licensure studies evaluating antibody persistence and protection after recombinant pertussis vaccination across sequential pregnancies will be needed to support such an application.

A pertussis-only formulation could have a positive impact on how vaccination is perceived and accepted in antenatal care, particularly in repeat pregnancies [49]. Repeat doses in subsequent pregnancies can be given without concerns of giving more tetanus and diphtheria toxoid vaccinations than necessary [10]. While there is no evidence that repeated Tdap vaccination within a short time period in women with closely spaced pregnancies might be associated with increased risk of severe reactions to tetanus toxoid, there may still be anxiety and a perceived risk of tetanus vaccination that may prevent pregnant women from getting (repeat) Tdap vaccinations [50,51]. Additionally, pertussis-only vaccine may avoid interference of maternally derived diphtheria antibodies with infant diphtheria vaccination and other vaccines containing (mutant) diphtheria toxin, such as pneumococcal conjugate vaccines (PCVs), and hence result in improved responses [52].

Of note, important evidence on the use of VacPertagen in pregnant women, including safety and effectiveness data, is arising from Thailand, where the vaccine has been marketed as Pertagen since 2016. To control a pertussis outbreak in the deep south of Thailand in 2023–2024, the Thai government introduced a three-pronged emergency campaign that included vaccinating pregnant women with VacPertagen. A subsequent retrospective study found that the use of VacPertagen in this emergency response was associated with a 95% effectiveness of protecting 0–3-month-old infants against symptomatic PCR-confirmed pertussis [53]; this aligns with the effectiveness of commonly used Tdap vaccines and the PT-only vaccine used in Denmark that reportedly ranges between 69 and 91% [16,54]. In response to the pertussis outbreak, in late 2024 the Thai government implemented a national maternal immunisation program using the recombinant pertussis-only vaccine. As the first country in the world using the new vaccine in its national maternal immunisation program, it will be interesting to watch data emerging from Thailand.

### 3.2. Healthcare Workers

Many countries have recommendations for healthcare workers to repeat pertussis vaccination every 5 or 10 years, despite the fact that anti-PT antibodies do not last for 10 years and likely shorter than 5 years after Tdap booster vaccination [41]. The recombinant pertussis vaccine induces anti-PT neutralising responses lasting for more than 5 years and would have the potential advantage of extending the period between repeat doses and avoiding unnecessary diphtheria and tetanus boosters. Additionally, because healthcare professionals are a trusted source of vaccine-related information and their endorsement of vaccination is a powerful factor in influencing community vaccination rates, a vaccine that provides longer protection could be beneficial to trust.

### 3.3. Other Adults

The burden of pertussis in adults is under recognised [55], with testing in the primary health care setting uncommon even when pertussis is suspected. Limited protection offered by Tdap vaccines in combination with under-recognition of the pertussis burden in adult populations have limited their use in this population [56]. However, the availability of a vaccine inducing longer-lasting antibodies could prompt a review of these recommendations and use by adults.

### 3.4. Adolescents

In Australia and New Zealand, a booster dose in secondary schools is in place. Replacing current pertussis vaccines with a vaccine offering longer-lasting immune protection in this age group would be of interest. However, it may need more investigation and consideration whether replacing a Tdap vaccine with a pertussis-only vaccine in this age group is suitable. In many high-income countries, national immunisation schedules do not follow the World Health Organisation recommendation of 6 doses of tetanus-diphtheria vaccines. While other vaccines in the childhood schedule containing tetanus or diphtheria toxin, such as *Haemophilus influenzae* type b, meningococcal ACWY, and PCVs, may contribute to boosting diphtheria and tetanus immunity, further data are needed to evaluate the potential role of a pertussis-only booster in adolescents, especially given recent outbreaks of diphtheria in remote and very remote areas of Australia [57].

## 4. Are Any Other New Pertussis Vaccines on the Horizon?

Candidates in clinical trials will not be available soon. A phase 1 trial has been completed for a candidate vaccine containing CpG 1018 adjuvant and demonstrated to be safe and induce slightly higher pertussis antibody responses than a licensed comparator vaccine [58]. In a controlled human infection model study, BPZE1, a live attenuated nasal pertussis vaccine candidate, was associated with less colonisation (42%) than licensed Tdap (67%) within a period of 60–120 days post-immunisation [59]. In a randomised controlled phase 2b trial including 6–17-year-olds, BPZE1 was associated with induction of mucosal secretory immunoglobulin A against whole-cell pertussis extract 29 days after immunisation [60]. A phase 3 trial is anticipated.

## 5. Discussion and Knowledge Gaps

Clinical trial results consistently indicate the new vaccine has a favorable immunogenicity profile in different target populations, including pregnant women. Post-marketing data from Thailand where the vaccine has been available for nearly a decade and was implemented in the National Immunisation Program to vaccinate pregnant women further support the safe use of the vaccine [61].

In view of ongoing pertussis outbreaks in Australia and New Zealand and other countries, replacing current Tdap boosters with the new vaccine may offer longer lasting protection and consequently contribute to lower case numbers and transmission in the community. The advantages and disadvantages of co-vaccination with Td need to be evaluated where Tdap vaccine is currently used for pertussis vaccination. Generally, pertussis immunity wanes faster than immunity against diphtheria and tetanus, and the availability of a pertussis-only vaccine now offers an option to give pertussis boosters without unnecessarily also repeating tetanus and diphtheria vaccination. This could be a preferred option for pregnant women, health care workers and other adults with persisting immunity against tetanus and diphtheria.

In contrast to Thailand, where the whole-cell pertussis vaccine continues to be used for primary childhood vaccination, Australia and New Zealand, like most other high-income countries, have changed to the exclusive use of acellular pertussis vaccines. A trial conducted in Australia demonstrated that booster vaccination with VacPertagen as compared with licensed Tdap resulted in significantly higher and longer-lasting anti-PT antibodies in young adults primed in childhood with either whole-cell or acellular pertussis vaccine [38]. Accordingly, a trial conducted in Switzerland including adolescents with a history of exclusive acellular pertussis vaccination reported significantly higher anti-PT antibody and B-cell memory responses after vaccination with VacPertagen versus Tdap [36]. These observations from populations including acellular primed subjects are highly relevant considering that adult cohorts, including pregnant women, are increasingly primed with acellular pertussis vaccine. First studies emerging from the US indicate that Tdap vaccination during pregnancy is associated with lower anti-PT antibody levels in pregnant women and infants when women were primed with acellular pertussis vaccine [62,63]. It will be important to understand if the reduction in antibodies transferred to infants is going to be associated with reduced vaccine effectiveness in infants when maternal antibodies wane below protective levels before infants have been able to establish their own immunity. The next question would be if VacPertagen avoids this risk by inducing sufficiently high anti-PT antibody levels in mothers to keep maternal antibodies in infants high enough over the first months of life to confer protection, even if mothers were primed with acellular pertussis vaccine.

There is very little data in general on the immunogenicity of pertussis vaccines and persistence of antibodies in older adults following booster vaccination. Yet, it is believed that antibody responses in the elderly may be lower and shorter-lived than in younger adults due to immunosenescence. Given the uncertainty around pertussis incidence, illness severity, initial vaccine effectiveness, and the duration of protection in adults and elderly, the US CDC concluded that vaccination with Tdap in this group may not be cost-effective [50]. Results from vaccination of a limited number of older adults with VacPertagen are encouraging, demonstrating higher and longer-persisting anti-PT antibodies compared with licensed Tdap [13]. Generating evidence on the burden of pertussis in adults and duration of immunity after vaccination with VacPertagen could potentially change opinions on the benefits of pertussis vaccination in adults.

Finally, there are no published data on the ability to boost immune memory induced by vaccination with VacPertagen but studies are underway. In a clinical trial conducted in Switzerland, repeat booster vaccination with VacPertagen is investigated in adults at a 6-month interval [64]. And in a study conducted in Thailand, responses to a booster dose of VacPertagen are studied in participants who received a dose of either VacPertagen or licensed Tdap vaccine 10 years earlier [65].

## 6. Conclusions

While the implementation of less reactogenic acellular pertussis vaccines has been critical in enhancing parental confidence and infant vaccine uptake, it has been documented for some time that immunity from acellular pertussis vaccines wanes more quickly than that from whole-cell pertussis vaccines. The first approval over two decades of a new pertussis vaccine for booster doses in the EU and the promise of a longer duration of effectiveness provide a timely opportunity to review pertussis vaccination recommendations, nationally and at a global level. In countries like Australia, New Zealand, the United Kingdom and most of the EU, where vaccine purchasing decisions are made based on cost-effectiveness criteria, use of the recombinant pertussis vaccine in national programmes will likely be accepted based on having higher effectiveness and/or duration if price is equivalent. Post-marketing data that will become available when the vaccine is adopted in more national programmes will need to demonstrate its acceptance and uptake, effectiveness, and safety at a population level. Currently available data on immunogenicity and safety are encouraging and point in a favourable direction.

## Data Availability

No new data were created or analyzed in this study. Data sharing is not applicable to this article.
